# Supplementary material for: Optogenetic dissection of basolateral amygdala contributions to intertemporal choice in young and aged rats
Source: eLife. 2019 Apr 24;8:e46174. doi: 10.7554/eLife.46174 (PMC6530979; doi:10.7554/eLife.46174)
Supplement: Transparent reporting form [file elife-46174-transrepform.docx]

***eLife’s* transparent reporting form**

We encourage authors to provide detailed information *within their submission* to facilitate the interpretation and replication of experiments. Authors can upload supporting documentation to indicate the use of appropriate reporting guidelines for health-related research (see [EQUATOR Network](http://www.equator-network.org/%20)), life science research (see the [BioSharing Information Resource](https://biosharing.org/)), or the [ARRIVE guidelines](http://www.plosbiology.org/article/info:doi/10.1371/journal.pbio.1000412) for reporting work involving animal research. Where applicable, authors should refer to any relevant reporting standards documents in this form.

If you have any questions, please consult our Journal Policies and/or contact us: [editorial@elifesciences.org](mailto:editorial@elifesciences.org).

**Sample-size estimation**

- You should state whether an appropriate sample size was computed when the study was being designed
- You should state the statistical method of sample size computation and any required assumptions
- If no explicit power analysis was used, you should describe how you decided what sample (replicate) size (number) to use

A power analysis (using GPower software) was conducted on preliminary data to determine the sample size necessary to detect effects of optogenetic inactivation on choice behavior, given the 2-factor repeated measures ANOVA design employed (described in the Methods, under Experimental Design and Statistical Analysis, Evaluation of BLA inactivation on intertemporal choice).

Please outline where this information can be found within the submission (e.g., sections or figure legends), or explain why this information doesn’t apply to your submission:

**Replicates**

- You should report how often each experiment was performed
- You should include a definition of biological versus technical replication
- The data obtained should be provided and sufficient information should be provided to indicate the number of independent biological and/or technical replicates
- If you encountered any outliers, you should describe how these were handled
- Criteria for exclusion/inclusion of data should be clearly stated
- High-throughput sequence data should be uploaded before submission, with a private link for reviewers provided (these are available from both GEO and ArrayExpress)

Please outline where this information can be found within the submission (e.g., sections or figure legends), or explain why this information doesn’t apply to your submission:

The numbers of rats of each age that were tested under each condition are provided in the Results section. There were no outliers in the datasets, and hence no data were excluded from the analyses (other than for reasons due to misplaced viral injections or cannula placements.

**Statistical reporting**

- Statistical analysis methods should be described and justified
- Raw data should be presented in figures whenever informative to do so (typically when N per group is less than 10)
- For each experiment, you should identify the statistical tests used, exact values of N, definitions of center, methods of multiple test correction, and dispersion and precision measures (e.g., mean, median, SD, SEM, confidence intervals; and, for the major substantive results, a measure of effect size (e.g., Pearson's r, Cohen's d)
- Report exact p-values wherever possible alongside the summary statistics and 95% confidence intervals. These should be reported for all key questions and not only when the p-value is less than 0.05.

Please outline where this information can be found within the submission (e.g., sections or figure legends), or explain why this information doesn’t apply to your submission:

The statistical analyses, including the specific tests used and exact group sizes, are described in Methods, Experimental Design and Statistical Analysis (as well as in the Results). Both effect sizes and exact p values are reported in the Results. Means and SEMs for all data are reported in the Figures and Tables. We did not present raw data in the figures, as we believe that the line graph format of much of the primary data would require a distracting number of lines to depict each subject. We do, however, provide all of the raw data that go into these graphs in table format in the “raw data” file.

(For large datasets, or papers with a very large number of statistical tests, you may upload a single table file with tests, Ns, etc., with reference to sections in the manuscript.)

**Group allocation**

- Indicate how samples were allocated into experimental groups (in the case of clinical studies, please specify allocation to treatment method); if randomization was used, please also state if restricted randomization was applied
- Indicate if masking was used during group allocation, data collection and/or data analysis

Rats were received from the vendor as either young or aged, and hence this was not an assignable condition. All other experiments employed within-subjects designs, such that rats were tested in both experimental (laser on) and control conditions. Note that the order in which the BLA was inactivated during different task epochs was randomized and counterbalanced across the two ages, and this is reported in the Methods, “Effects of optogenetic inhibition during specific task epochs”. The obvious differences in appearance between young and aged rats, as well as the obvious presence or absence of the laser light used for optogenetic inactivation, made it impossible to mask these conditions during data collection. Histological analyses and decisions on inclusion/exclusion of rats based on viral expression and cannula placements were conducted masked to the rats’ behavioral performance (this latter point is described in the Methods, Vector Expression and Cannula Placement Histology)

Please outline where this information can be found within the submission (e.g., sections or figure legends), or explain why this information doesn’t apply to your submission:

**Additional data files (“source data”)**

- We encourage you to upload relevant additional data files, such as numerical data that are represented as a graph in a figure, or as a summary table
- Where provided, these should be in the most useful format, and they can be uploaded as “Source data” files linked to a main figure or table
- Include model definition files including the full list of parameters used
- Include code used for data analysis (e.g., R, MatLab)
- Avoid stating that data files are “available upon request”

Please indicate the figures or tables for which source data files have been provided:

The raw data that are depicted in summary form in Figures 4-8 and that were used for statistical analyses are provided in source data files corresponding to each Figure. The source data for the electrophysiological data analyses are also provided.
